# Peri-procedural and early body temperature trajectories after endovascular thrombectomy and clinical outcomes in intensive care unit patients with acute ischemic stroke: a retrospective cohort study

**DOI:** 10.3389/fneur.2026.1941980

**Published:** 2026-09-25

**Authors:** Xidong Wu, Fujin Chen, Yujie Pan, Xiaobo Wang, Jun Sun

**Affiliations:** 1 Department of Neurosurgery, Wenzhou Central Hospital Affiliated to Wenzhou Medical University, Wenzhou, Zhejiang, China; 2 Department of Critical Care Medicine, Wenzhou Central Hospital Affiliated to Wenzhou Medical University, Wenzhou, Zhejiang, China

**Keywords:** acute ischemic stroke, body temperature, endovascular thrombectomy, hyperthermia, mortality, seizure, trajectory analysis

## Abstract

**Background:**

Body temperature after endovascular thrombectomy (EVT) is often summarized by single measurements or fever thresholds, which do not capture temporal patterns. We examined peri-procedural and early post-EVT temperature trajectories and their associations with 90-day outcomes in ICU patients with acute ischemic stroke.

**Methods:**

This single-center retrospective cohort included 237 ICU patients undergoing EVT. Axillary temperature was recorded at nine prespecified time points from EVT initiation through 72 h. Patient-specific cubic curves were clustered using Gaussian mixture models. The primary outcome was 90-day unfavorable functional outcome (modified Rankin Scale 3–6); secondary outcomes included 90-day mortality and post-stroke seizures. Multivariable models adjusted for prespecified baseline and procedural covariates.

**Results:**

Four trajectories were identified: stable normothermia (*n* = 89), early transient temperature rise (*n* = 49), delayed fever (*n* = 32), and persistent hyperthermia (*n* = 67). Unfavorable functional outcome occurred in 143 patients (60.3%) and 60 (25.3%) died by 90 days. The global trajectory-class test was significant for mortality (*p* = 0.019) but not for unfavorable functional outcome (*p* = 0.088) or post-exposure seizure (*p* = 0.126). Compared with stable normothermia, persistent hyperthermia was associated with higher 90-day mortality (adjusted OR 4.51, 95% CI 1.65–12.34). This association was attenuated after adjustment for early antipyretic use (adjusted OR 3.02, 95% CI 0.98–9.32; global *p* = 0.241) and after exclusion of patients with infection within 72 h (adjusted OR 3.92, 95% CI 1.18–12.97; global *p* = 0.066). Seizure analyses were exploratory.

**Conclusion:**

Greater temperature exposure was associated with worse outcomes, consistent with previous work on post-stroke fever. Persistent hyperthermia was associated with mortality in the primary analysis, but the association weakened in key sensitivity analyses. Trajectory classification did not establish added prognostic value over simpler temperature metrics and should be considered exploratory rather than a new prognostic tool.

## 1 Introduction

Endovascular thrombectomy (EVT) has substantially improved the treatment of acute ischemic stroke caused by large-vessel occlusion (1, 2). However, successful reperfusion does not always translate into favorable clinical recovery (3). A considerable proportion of patients remain severely disabled, die, or develop neurological complications despite technically successful recanalization. This mismatch highlights the importance of peri-procedural and early post-EVT physiological changes, particularly among patients requiring intensive care (4).

Body temperature is one of the most routinely monitored physiological variables in the ICU (4). Fever and hyperthermia are common after acute ischemic stroke and may occur in association with neurological injury, infection, inflammation, hemorrhagic transformation, impaired consciousness, mechanical ventilation, and systemic critical illness (5–8). In post-EVT ICU patients, temperature elevation may therefore reflect a complex interaction between brain injury, systemic complications, and treatment responses rather than a simple abnormality in a vital sign (9–11).

Most previous studies have evaluated post-stroke temperature using admission or peak temperature, binary fever definitions, fever burden, or the number of fever episodes within a fixed window (12, 13). These measures are clinically intuitive but may obscure temporal differences. A brief early temperature rise, delayed fever, and sustained hyperthermia may reflect different combinations of neurological injury, systemic inflammation, infection, organ support, and treatment response.

Trajectory analysis of repeated measurements can preserve temporal information that is not represented by a single summary value (14). In ICU patients undergoing EVT, frequent temperature monitoring provides an opportunity to characterize heterogeneous peri-procedural and early post-procedural patterns. However, the associations of these patterns with clinically meaningful outcomes have not been well characterized.

We used body temperature measurements obtained from EVT initiation through 72 h to identify model-based temperature trajectories in ICU patients with acute ischemic stroke. We then examined associations with 90-day functional outcome, 90-day mortality, post-exposure seizure after the 72-h temperature window, and seizure during days 8–90 after stroke (15). The seizure outcomes were exploratory because temperature elevation and seizures may share determinants—including stroke severity, cortical injury, hemorrhagic transformation, and inflammation—without implying a direct causal relationship. Continuous temperature-burden metrics were evaluated as complementary secondary exposures.

## 2 Materials and methods

### 2.1 Study design and population

This single-center retrospective cohort study included consecutive adult ICU patients with acute ischemic stroke who underwent EVT at Wenzhou Central Hospital Affiliated to Wenzhou Medical University between June 1, 2020, and December 31, 2025. ICU admission after EVT was selective rather than routine. General anesthesia alone was not an indication for ICU admission. Patients were generally admitted when they required ongoing airway or ventilatory support, intensive neurological monitoring because of impaired consciousness or severe neurological deficits, hemodynamic support, close monitoring after high-risk posterior-circulation stroke, or management of relevant peri-procedural complications. ICU bed availability could alter disposition; when beds were unavailable, selected patients needing continued ventilatory support could be managed in an enhanced-monitoring area of the neurosurgical ward and were classified as non-ICU. Mechanical ventilation was defined as invasive ventilatory support continuing after EVT; intraprocedural intubation for general anesthesia was not counted if extubation occurred at the end of the procedure. The same definition was used in both groups. Clinically stable patients suitable for routine stroke-unit care could be admitted directly to the stroke unit after EVT. Eligible ICU patients were required to have axillary temperature measurements at all nine prespecified time points (0, 2, 6, 12, 24, 36, 48, 60, and 72 h after EVT initiation).

Patients were excluded if key baseline, procedural, or 90-day outcome data were unavailable, if an eligible axillary temperature measurement was missing at any of the nine prespecified time points, or if major time variables were internally inconsistent. Temperature series were incomplete when measurements were interrupted by transfer to another department, death, or self-discharge against medical advice before completion of the 72-h exposure window, or when no eligible value was available within the ±2-h window around at least one prespecified time point despite ongoing hospitalization. Thus, a patient with frequent routine measurements could still lack a complete nine-point analytical series. Missing temperature values were not imputed. Accordingly, trajectory assignment and the mortality analyses were conditional on survival and continued observation through 72 h. Primary models used complete-case data; all 237 included patients had complete data for the prespecified primary covariates. For the exploratory day 8–90 seizure analysis, the risk set was restricted to patients with documented survival and evaluable follow-up at the beginning of day 8. Patients who died in hospital or were discharged in a moribund condition before day 8 were excluded from this risk set.

The study was approved by the Ethics Committee of Wenzhou Central Hospital Affiliated to Wenzhou Medical University (approval number: L2026-01-020) and was conducted in accordance with the Declaration of Helsinki. The requirement for informed consent was waived because of the retrospective design and use of de-identified clinical data.

### 2.2 Temperature measurements and trajectory identification

EVT initiation was defined as arterial puncture and served as time zero. The exposure window therefore spanned EVT initiation through 72 h and was considered peri-procedural and early post-EVT rather than exclusively post-procedural. Body temperature was measured by the axillary route throughout the study. Routine clinical temperature monitoring was more frequent than the nine measurements selected for analysis. The sampling grid at 0, 2, 6, 12, 24, 36, 48, 60, and 72 h was prespecified to capture rapid peri-procedural changes more densely during the first 12–24 h, followed by 12-h sampling for the remainder of the exposure window. A measurement obtained within 2 h before or after each prespecified time point was eligible; when multiple measurements fell within the matching window, the one closest to the target time was used.

Each patient’s 0- to 72-h temperature profile was summarized with a cubic polynomial function of time. Measurement time was divided by 72 h to scale it to the 0–1 interval, and the patient-specific intercept, linear, quadratic, and cubic coefficients were standardized using StandardScaler. Gaussian mixture models with one to five candidate classes were fitted using unrestricted full covariance matrices, 10 initializations, covariance regularization of 1 × 10^−5^, a maximum of 500 expectation–maximization iterations, and a fixed random seed. Candidate solutions were evaluated using the Akaike and Bayesian information criteria, entropy, minimum class proportion, posterior classification probability, convergence, parsimony, and clinical interpretability. Outcomes were not used to identify classes. Each patient was assigned to the class with the highest posterior probability, and class labels were assigned after inspection of the mean curves. Because temperature-lowering interventions could affect subsequent measurements within the same 72-h window, we interpreted the classes as temperature patterns observed under routine care, recognizing that later measurements may have been modified by treatment.

Secondary continuous exposures were derived from the nine selected temperature values over the 0–72-h window. Peak temperature was defined as the maximum selected value, and temperature variability as the within-patient standard deviation of the selected values. Between adjacent prespecified time points, temperature was assumed to change linearly. Threshold-crossing times were estimated by linear interpolation; fever burden was calculated as the total interpolated duration with temperature ≥37.5 °C. Temperature AUC above 37.5 °C was calculated using trapezoidal integration of the positive temperature excess, max[T(t) − 37.5, 0], and was expressed in degree-hours. In regression models, fever burden was scaled per 6 h, temperature AUC per 10 °C·h, and temperature variability per 0.5 °C.

### 2.3 Outcomes and follow-up

The primary outcome was unfavorable functional outcome at 90 days, defined as a modified Rankin Scale score of 3–6.

Secondary outcomes were 90-day mortality, post-exposure seizure from 72 h after EVT initiation through day 7 after stroke onset, and seizure during days 8–90 after stroke onset. The first seizure window began at 72 h to avoid temporal overlap with the 0–72-h temperature exposure; it therefore does not cover the full conventional acute symptomatic seizure period. Ninety-day mortality was defined as a 90-day modified Rankin Scale score of 6; patients who died before the 90-day assessment were assigned a score of 6. Ninety-day functional status and vital status were obtained from inpatient records, outpatient follow-up records, and structured telephone follow-up documented in the medical record. When more than one source was available, records were cross-checked and the most complete assessment closest to day 90 was used. Seizure outcomes were ascertained from inpatient and outpatient neurological documentation, electroencephalography when available, antiseizure medication records, and follow-up documentation. The day 8–90 seizure analysis was restricted to patients with documented survival and evaluable follow-up at the beginning of day 8. Outcome data were not imputed.

### 2.4 Covariates

Prespecified baseline and procedural covariates were age, sex, baseline National Institutes of Health Stroke Scale score, pretreatment ischemic core volume, onset-to-recanalization or procedure-end time, successful recanalization, and anterior-circulation involvement. Pretreatment computed tomography perfusion was performed after hospital presentation and before EVT, generally within several hours of the procedure, and processed using Dr. Wise-CTP (version V3.0.0.1). Ischemic core was defined as tissue with relative cerebral blood flow <30% of normally perfused brain tissue. The available technical records did not specify a minimum lesion-size or cluster-size threshold. All 237 included patients underwent pretreatment CTP; 118 (49.8%) had a software-reported core volume of 0 mL. Recorded 0-mL values were direct software outputs rather than missing or imputed values. ASPECTS was included as an adjustment covariate only in the corresponding sensitivity analysis restricted to anterior-circulation stroke. Mechanical ventilation, infection within 72 h, symptomatic intracranial hemorrhage, early neurological deterioration (END), and temperature-management measures were treated as early clinical or treatment-response variables rather than baseline confounders.

Successful recanalization was defined as a final modified Thrombolysis in Cerebral Infarction score of 2b–3. EVT initiation corresponded to arterial puncture. For successfully recanalized patients, treatment time was measured from stroke onset to angiographic recanalization; for those without successful recanalization, it was measured from stroke onset to procedure end. Standard procedural intervals, including onset-to-puncture, puncture-to-recanalization, and onset-to-recanalization times, were also summarized descriptively. Anterior-circulation involvement was derived from the responsible vessel, with vertebral and basilar artery occlusions classified as posterior circulation. Infection within 72 h included clinically documented pneumonia or another early infection.

Additional baseline descriptors included pre-stroke mRS, hypertension, diabetes mellitus, atrial fibrillation, dyslipidemia, previous stroke or transient ischemic attack, current or former smoking, TOAST-based stroke etiology, and responsible-vessel occlusion site. These variables were reported descriptively and were not part of the prespecified primary adjustment set.

Active temperature management within 72 h was defined as a documented protocolized temperature-control intervention, antipyretic therapy, or physical cooling. Because these variables and other early complications could occur during or after formation of the temperature trajectory, they were evaluated only in sensitivity or exploratory analyses.

Covariates were selected before outcome modeling according to clinical relevance, temporal availability, and their potential associations with both temperature trajectory and clinical outcomes.

### 2.5 Statistical analysis

Continuous variables are reported as medians with interquartile ranges and categorical variables as counts with percentages. Across-class comparisons used Kruskal–Wallis tests for continuous variables and chi-square or Fisher exact tests for categorical variables, as appropriate.

Multivariable logistic regression was used to estimate odds ratios (ORs) with 95% confidence intervals (CIs). Stable normothermia was the reference class in categorical analyses. Likelihood-ratio tests were used to assess the overall contribution of the categorical trajectory variable before individual class contrasts were interpreted. The unfavorable-outcome model adjusted for age, sex, baseline NIHSS, ischemic core volume, treatment time, successful recanalization, and anterior-circulation involvement. The mortality model used the same set without sex, whereas the post-exposure seizure model adjusted for age, baseline NIHSS, treatment time, and anterior-circulation involvement. When the global trajectory-class test was not statistically significant, class-specific contrasts were treated as exploratory.

Because only 17 seizures occurred during days 8–90, this analysis was considered exploratory. Persistent hyperthermia was compared with all non-persistent trajectories in a parsimonious model adjusting for age, baseline NIHSS, treatment time, post-exposure seizure from 72 h through day 7, and anterior-circulation involvement. The analysis included 223 patients with documented survival and evaluable follow-up at the beginning of day 8. Given the small number of events, non-systematic EEG assessment, and competing mortality, we did not fit a more extensive etiologic model.

An exploratory ordered-trend analysis arranged the four classes by increasing fever burden and modeled the resulting ordinal variable per one-class increase. This analysis was complementary to, and did not replace, the categorical class analysis.

Continuous temperature-burden metrics were evaluated using outcome-specific covariate sets consistent with the corresponding trajectory analyses.

Sensitivity analyses for the two 90-day outcomes retained the categorical trajectory specification and reported the persistent-hyperthermia versus stable-normothermia contrast together with the global trajectory-class test. Models additionally adjusted for mechanical ventilation and infection within 72 h, active temperature management, early antipyretic use, physical cooling, END, or pre-stroke mRS; excluded patients with infection within 72 h; restricted the analysis to anterior-circulation stroke with ASPECTS adjustment; or excluded class assignments with posterior probability <0.80. Given the high proportion of software-reported 0-mL core estimates, additional sensitivity analyses entered ischemic core categorically (0, >0–30, and >30 mL) or omitted it from the adjustment set. Exploratory ordered-trend sensitivity analyses are reported in the Supplementary Materials. Exploratory subgroup analyses for the primary outcome were limited to age, successful recanalization, and anterior-circulation involvement, with interaction terms used to assess effect modification.

All tests were two-sided. *p* < 0.05 was used as a conventional threshold, but secondary, ordered-trend, subgroup, and seizure analyses were considered exploratory and no formal adjustment for multiple testing was applied. Statistical analyses were performed using Python 3.13 with statsmodels and scikit-learn. Figures were generated using Matplotlib. The study was reported in accordance with the Strengthening the Reporting of Observational Studies in Epidemiology (STROBE) statement.

## 3 Results

### 3.1 Cohort characteristics and trajectory identification

A total of 468 patients who underwent EVT were screened; 336 (71.8%) were admitted to the ICU after EVT and 132 were managed outside the ICU. During the same study period, 22,714 patients were hospitalized with acute ischemic stroke, of whom 468 (2.1%) underwent EVT. Compared with the 132 non-ICU patients, ICU-admitted patients had greater neurological severity [median baseline NIHSS 18 (IQR 13–23) vs. 12 (IQR 8–15); signed SMD 1.002], more frequent general anesthesia (94.3% vs. 70.5%; SMD 0.661) and mechanical ventilation (60.7% vs. 17.4%; SMD 0.990), and higher 90-day mortality among patients with evaluable follow-up (83/313 [26.5%] vs. 12/132 [9.1%]). Successful recanalization was similar between groups (75.3% vs. 79.5%; SMD − 0.102) (Supplementary Table S5). Among the 336 ICU patients, 99 were excluded: 60 had insufficient serial temperature data during the first 72 h, 23 lacked 90-day mRS or follow-up data, and 16 had incomplete key baseline or procedural variables. The final analytic cohort comprised 237 patients (Supplementary Figure S1). Compared with included patients, the 99 excluded ICU patients had higher baseline NIHSS [19 (15–24) vs. 18 (12–22); SMD − 0.316] and lower successful recanalization (66.7% vs. 78.9%; SMD 0.278). The 60 patients excluded specifically for incomplete temperature observations had higher baseline NIHSS [20.5 (16–27) vs. 18 (12–22); SMD − 0.526], more frequent mechanical ventilation (73.3% vs. 58.2%; SMD − 0.322), and included 11 patients (18.3%) who died before completion of the 72-h window. Other reasons for incomplete temperature observation included early transfer out of the ICU, self-discharge, and incomplete routine documentation. Incomplete observation therefore occurred at both ends of the clinical spectrum: some patients deteriorated or died early, whereas others improved sufficiently for transfer out of the ICU before 72 h (Supplementary Tables S5, S6). Candidate models with one to five classes were fitted. The four-class solution was selected on the basis of model fit, posterior classification, class size, convergence, parsimony, and clinical interpretability. Although the five-class solution had a lower AIC and a marginally lower BIC than the four-class solution (BIC 147.7 vs. 149.1), its additional class contained only one patient (0.4%). In a *post hoc* initialization-sensitivity analysis across 30 random-state values, the four-class patient partition used in the primary analysis was reproduced exactly in 28 of 30 fits, whereas five-class solutions were more sensitive to initialization and produced a class of five or fewer patients in 11 of 30 fits (Supplementary Table S7). The four-class solution was therefore retained as the primary model, although external validation remains necessary.

The four trajectories were stable normothermia (*n* = 89, 37.6%), early transient temperature rise (*n* = 49, 20.7%), delayed fever (*n* = 32, 13.5%), and persistent hyperthermia (*n* = 67, 28.3%). Average posterior probabilities were 0.974, 0.973, 0.988, and 0.909, respectively (Figure 1; Supplementary Figure S2; Supplementary Tables S1, S2).

**Figure 1:**
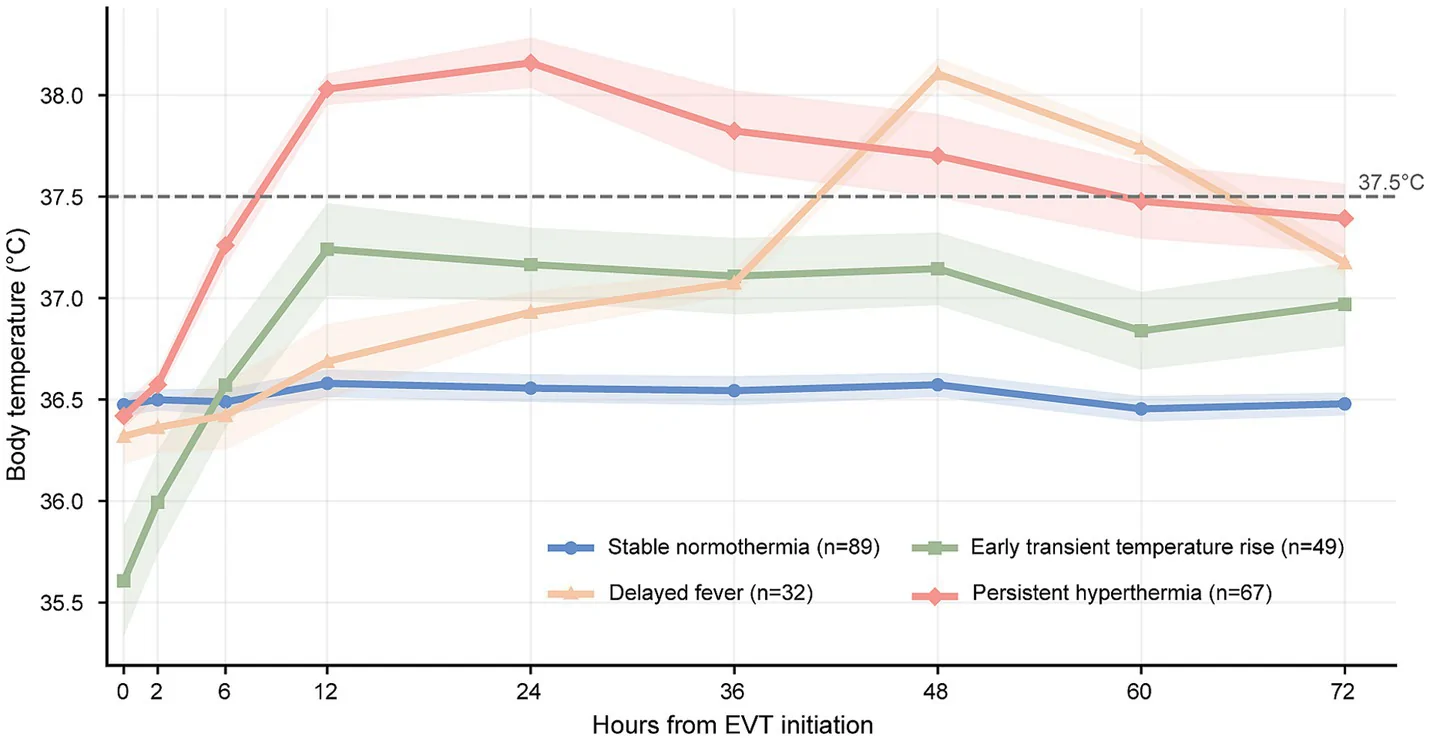
Peri-procedural and early post-EVT temperature trajectories. Mean body temperature with 95% confidence intervals is shown for the four model-based classes from EVT initiation through 72 h. The dashed line indicates 37.5 °C. EVT, endovascular thrombectomy.

### 3.2 Clinical and treatment characteristics

Table 1 summarizes baseline, imaging, procedural, clinical, and treatment characteristics, including pre-stroke functional status, vascular risk factors, TOAST-based stroke etiology, occlusion site, standard procedural time intervals, and the full final mTICI distribution. Baseline NIHSS was higher in the delayed-fever and persistent-hyperthermia classes than in the stable-normothermia class. ASPECTS was summarized only among the 186 patients with anterior-circulation stroke. Early infection was more frequent in the delayed-fever and persistent-hyperthermia classes, whereas mechanical ventilation was most frequent in the early transient temperature-rise class. Successful recanalization rates were similar across classes. Among the 186 patients with anterior-circulation stroke, 86 (46.2%) had a software-reported 0-mL core; the between-class difference in core volume remained significant (*p* = 0.002; Supplementary Table S9).

**Table 1:** Clinical and treatment characteristics according to model-based temperature trajectory classes.

Characteristic	Overall (*n* = 237)	Stable normothermia (*n* = 89)	Early transient temperature rise (*n* = 49)	Delayed fever (*n* = 32)	Persistent hyperthermia (*n* = 67)	*p-*value
Age, years	70.0 (60.0–78.0)	68.0 (60.0–75.0)	70.0 (55.0–78.0)	74.0 (58.2–81.5)	71.0 (63.5–80.0)	0.281
Male sex	163 (68.8)	61 (68.5)	38 (77.6)	24 (75.0)	40 (59.7)	0.179
Pre-stroke mRS	0 (0–1)	0 (0–1)	0 (0–1)	0 (0–0)	0 (0–1)	0.907
Hypertension	119 (50.2)	41 (46.1)	25 (51.0)	17 (53.1)	36 (53.7)	0.786
Diabetes mellitus	60 (25.3)	27 (30.3)	11 (22.4)	4 (12.5)	18 (26.9)	0.234
Atrial fibrillation	90 (38.0)	29 (32.6)	22 (44.9)	9 (28.1)	30 (44.8)	0.193
Dyslipidemia	31 (13.1)	12 (13.5)	7 (14.3)	5 (15.6)	7 (10.4)	0.881
Previous stroke/TIA	55 (23.2)	25 (28.1)	6 (12.2)	4 (12.5)	20 (29.9)	0.042
Current or former smoking	90 (38.0)	35 (39.3)	20 (40.8)	13 (40.6)	22 (32.8)	0.781
Baseline NIHSS	18.0 (12.0–22.0)	14.0 (11.0–20.0)	17.0 (11.0–21.0)	21.0 (16.8–25.2)	21.0 (15.0–25.0)	<0.001
ASPECTS, anterior circulation only	9.0 (7.0–10.0)	10.0 (7.0–10.0)	8.0 (6.0–10.0)	7.0 (6.0–10.0)	10.0 (7.0–10.0)	0.050
Ischemic core volume, mL	0.3 (0.0–23.0)	0.0 (0.0–15.0)	17.0 (0.0–32.0)	5.0 (0.0–27.2)	0.0 (0.0–15.0)	0.002
Stroke etiology (TOAST-based)						0.166
LAA	102 (43.0)	40 (44.9)	18 (36.7)	20 (62.5)	24 (35.8)	
Cardioembolism	95 (40.1)	31 (34.8)	23 (46.9)	9 (28.1)	32 (47.8)	
Other/undetermined	40 (16.9)	18 (20.2)	8 (16.3)	3 (9.4)	11 (16.4)	
Occlusion site						0.362
Carotid	55 (23.2)	22 (24.7)	10 (20.4)	6 (18.8)	17 (25.4)	
MCA	122 (51.5)	48 (53.9)	29 (59.2)	15 (46.9)	30 (44.8)	
ACA	9 (3.8)	0 (0.0)	2 (4.1)	2 (6.2)	5 (7.5)	
Basilar/vertebrobasilar	41 (17.3)	15 (16.9)	6 (12.2)	9 (28.1)	11 (16.4)	
Vertebral	10 (4.2)	4 (4.5)	2 (4.1)	0 (0.0)	4 (6.0)	
Bridging thrombolysis	129 (54.4)	61 (68.5)	23 (46.9)	18 (56.2)	27 (40.3)	0.003
General anesthesia	228 (96.2)	87 (97.8)	49 (100.0)	30 (93.8)	62 (92.5)	0.138
Onset-to-puncture time, min	300 (204–433)	330 (217–498)	255 (165–345)	300 (214–428)	303 (207–441)	0.168
Puncture-to-recanalization time, min*	62 (44–88)	50 (42–75)	88 (58–109)	62 (47–80)	63 (42–77)	<0.001
Onset-to-recanalization time, min*	365 (259–469)	356 (243–492)	355 (259–430)	353 (273–456)	389 (282–509)	0.745
Onset-to-recanalization or procedure-end time, min	384 (271–518)	393 (280–558)	365 (261–436)	387 (286–525)	390 (279–509)	0.718
Successful recanalization	187 (78.9)	67 (75.3)	41 (83.7)	26 (81.2)	53 (79.1)	0.687
Final mTICI grade						0.848
0	8 (3.4)	3 (3.4)	2 (4.1)	1 (3.1)	2 (3.0)	
1	16 (6.8)	7 (7.9)	3 (6.1)	2 (6.2)	4 (6.0)	
2a	26 (11.0)	12 (13.5)	3 (6.1)	3 (9.4)	8 (11.9)	
2b	65 (27.4)	25 (28.1)	12 (24.5)	6 (18.8)	22 (32.8)	
2c	36 (15.2)	14 (15.7)	6 (12.2)	4 (12.5)	12 (17.9)	
3	86 (36.3)	28 (31.5)	23 (46.9)	16 (50.0)	19 (28.4)	
Anterior circulation	186 (78.5)	70 (78.7)	41 (83.7)	23 (71.9)	52 (77.6)	0.650
Mechanical ventilation	138 (58.2)	35 (39.3)	47 (95.9)	18 (56.2)	38 (56.7)	<0.001
Active temperature management within 72 h	129 (54.4)	28 (31.5)	48 (98.0)	16 (50.0)	37 (55.2)	<0.001
Antipyretic use within 24 h	73 (30.8)	5 (5.6)	18 (36.7)	10 (31.2)	40 (59.7)	<0.001
Physical cooling within 24 h	84 (35.4)	8 (9.0)	22 (44.9)	11 (34.4)	43 (64.2)	<0.001
Infection within 72 h	70 (29.5)	15 (16.9)	15 (30.6)	15 (46.9)	25 (37.3)	0.004
Symptomatic intracranial hemorrhage	22 (9.3)	6 (6.7)	5 (10.2)	8 (25.0)	3 (4.5)	0.008
Early neurological deterioration	70 (29.5)	20 (22.5)	13 (26.5)	13 (40.6)	24 (35.8)	0.138
Peak temperature, °C	37.8 (36.9–38.3)	36.8 (36.7–37.0)	37.8 (37.5–38.3)	38.2 (38.0–38.3)	38.4 (38.1–38.8)	<0.001
Fever burden ≥37.5 °C, h	13.9 (0.0–25.3)	0.0 (0.0–0.0)	12.0 (2.3–24.0)	24.6 (22.9–25.8)	34.0 (21.8–64.9)	<0.001

Continuous variables are presented as median (interquartile range) and categorical variables as *n* (%). *p*-values were calculated using Kruskal–Wallis tests for continuous variables and chi-square or Fisher exact tests for categorical variables, as appropriate. ASPECTS is summarized only for anterior-circulation stroke (*n* = 186). Pretreatment ischemic core volume was derived from CT perfusion performed after hospital presentation and before EVT and processed using Dr. Wise-CTP; ischemic core was defined as tissue with relative cerebral blood flow <30% of normally perfused brain tissue. Recorded 0-mL values were direct software outputs rather than missing or imputed values. EVT initiation corresponded to arterial puncture. *Puncture-to-recanalization and onset-to-recanalization times are summarized among successfully recanalized patients only (*n* = 187). Active temperature management refers to documented temperature-control measures within 72 h after EVT initiation. ASPECTS, Alberta Stroke Program Early CT Score; END, early neurological deterioration; LAA, large-artery atherosclerosis; MCA, middle cerebral artery; ACA, anterior cerebral artery; mTICI, modified Thrombolysis in Cerebral Infarction; NIHSS, National Institutes of Health Stroke Scale; TIA, transient ischemic attack. For multi-level categorical variables, the p-value shown on the parent row refers to the overall comparison of the full category distribution.

Active temperature management differed across classes and was most frequent in the early transient temperature-rise class. Antipyretic use and physical cooling were more common in persistent hyperthermia than in stable normothermia; these variables were evaluated as treatment-response variables in sensitivity analyses. The distribution of fever burden across trajectory classes is shown in Supplementary Figure S3.

### 3.3 Clinical outcomes

At 90 days, 143 patients (60.3%) had an unfavorable functional outcome and 60 (25.3%) had died. The full 90-day mRS distribution was 21 (8.9%), 42 (17.7%), 31 (13.1%), 34 (14.3%), 31 (13.1%), 18 (7.6%), and 60 (25.3%) for mRS scores 0 through 6, respectively (Table 2). Post-exposure seizure from 72 h through day 7 occurred in 33 patients (13.9%). Seizure during days 8–90 occurred in 17 of 223 patients with documented survival and evaluable follow-up at day 8 (7.6%).

**Table 2:** Clinical outcomes according to model-based temperature trajectory classes.

Outcome	Overall (*n* = 237)	Stable normothermia (*n* = 89)	Early transient temperature rise (*n* = 49)	Delayed fever (*n* = 32)	Persistent hyperthermia (*n* = 67)	*p-*value
90-day unfavorable outcome (mRS 3–6)	143 (60.3)	38 (42.7)	34 (69.4)	25 (78.1)	46 (68.7)	<0.001
90-day mortality	60 (25.3)	8 (9.0)	16 (32.7)	11 (34.4)	25 (37.3)	<0.001
90-day mRS distribution						0.017
mRS 0	21 (8.9)	11 (12.4)	3 (6.1)	1 (3.1)	6 (9.0)	
mRS 1	42 (17.7)	23 (25.8)	6 (12.2)	4 (12.5)	9 (13.4)	
mRS 2	31 (13.1)	17 (19.1)	6 (12.2)	2 (6.2)	6 (9.0)	
mRS 3	34 (14.3)	11 (12.4)	8 (16.3)	4 (12.5)	11 (16.4)	
mRS 4	31 (13.1)	13 (14.6)	7 (14.3)	5 (15.6)	6 (9.0)	
mRS 5	18 (7.6)	6 (6.7)	3 (6.1)	5 (15.6)	4 (6.0)	
mRS 6	60 (25.3)	8 (9.0)	16 (32.7)	11 (34.4)	25 (37.3)	
Post-exposure seizure (72 h to day 7)	33 (13.9)	8 (9.0)	4 (8.2)	5 (15.6)	16 (23.9)	0.032
Seizure during days 8–90 after stroke	17/223 (7.6)	2/89 (2.2)	2/46 (4.3)	3/28 (10.7)	10/60 (16.7)	0.008

Outcome events are presented as *n* (%) except seizure during days 8–90, which is shown as events/eligible patients (%). The full 90-day mRS distribution is provided as *n* (%) for scores 0–6. The day 8–90 seizure denominator includes patients with documented survival and evaluable follow-up at the beginning of day 8 (*n* = 223); patients who died in hospital or were discharged in a moribund condition before day 8 were excluded. The 72 h–day 7 seizure outcome begins after completion of the temperature exposure window and therefore does not represent the full conventional acute symptomatic seizure period. *p*-values were calculated using chi-square or Fisher exact tests, as appropriate. mRS, modified Rankin Scale. For the 90-day mRS distribution, the *p*-value shown on the parent row refers to the overall comparison across mRS categories 0–6.

Crude outcome rates differed across trajectory classes (Table 2 and Figure 2). Unfavorable functional outcome occurred in 42.7, 69.4, 78.1, and 68.7% of the stable-normothermia, early transient temperature-rise, delayed-fever, and persistent-hyperthermia classes, respectively. Corresponding 90-day mortality rates were 9.0, 32.7, 34.4, and 37.3%. Post-exposure seizure occurred in 9.0, 8.2, 15.6, and 23.9%, respectively, and seizure during days 8–90 occurred in 2.2, 4.3, 10.7, and 16.7% of eligible patients, respectively.

**Figure 2:**
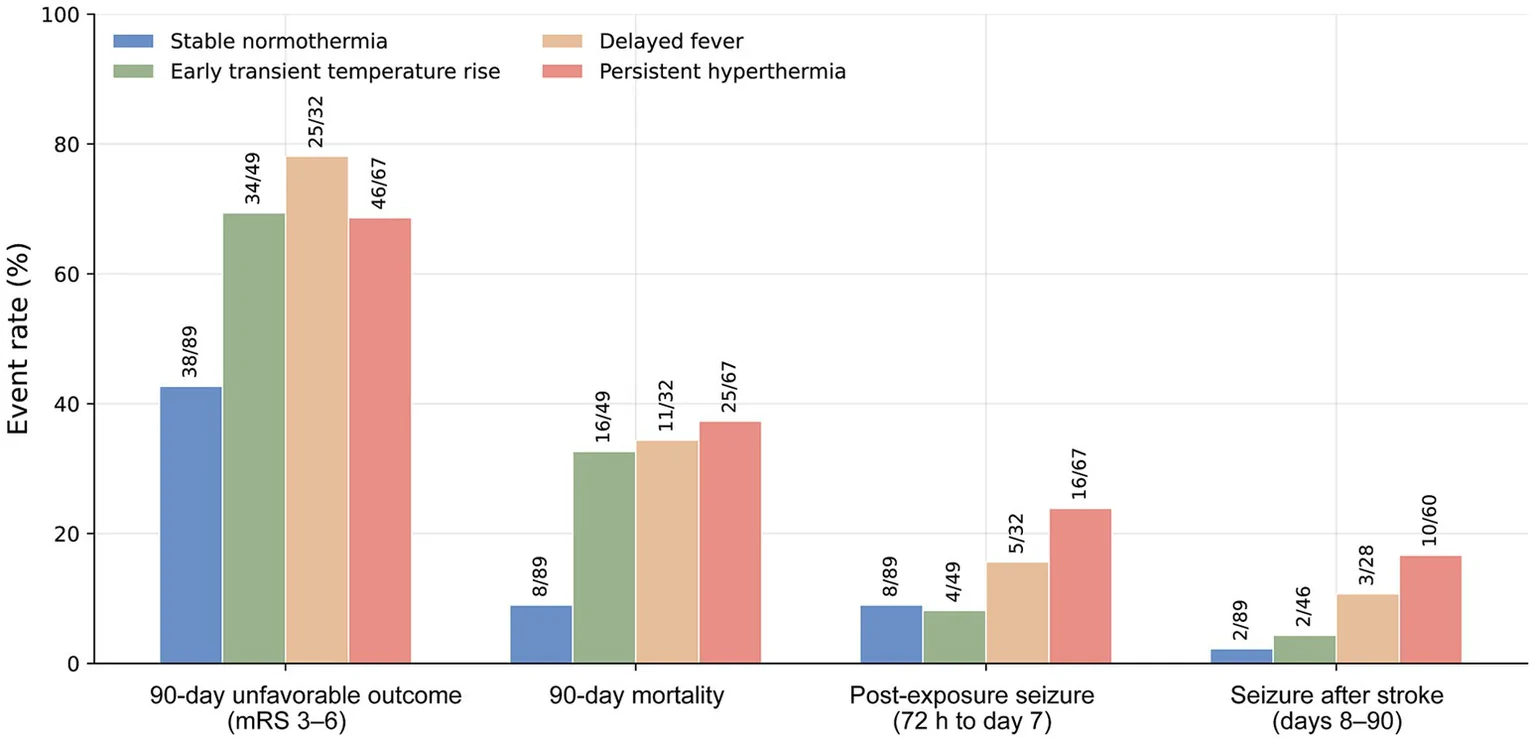
Crude outcome rates by temperature trajectory class. Bars show crude event rates for 90-day unfavorable functional outcome, 90-day mortality, post-exposure seizure from 72 h after EVT initiation through day 7, and seizure after stroke (days 8–90). Labels show events/eligible patients. For the day 8–90 seizure outcome, denominators were restricted to patients with documented survival and evaluable follow-up at day 8 (*n* = 223). Seizure outcomes were exploratory.

### 3.4 Adjusted associations

The overall trajectory-class test was not statistically significant for the primary unfavorable functional outcome (global *p* = 0.088), so class-specific contrasts were treated as exploratory. Compared with stable normothermia, persistent hyperthermia yielded an adjusted OR of 2.36 (95% CI 1.07–5.20; *p* = 0.032). Estimates for early transient temperature rise (adjusted OR 2.00, 95% CI 0.84–4.76; *p* = 0.119) and delayed fever (adjusted OR 2.60, 95% CI 0.90–7.54; *p* = 0.079) were imprecise. The exploratory ordered-trend estimate was 1.35 per class increase (95% CI 1.04–1.75; *p* = 0.023).

For 90-day mortality, the overall trajectory-class test was significant (global *p* = 0.019). Persistent hyperthermia was associated with higher mortality than stable normothermia (adjusted OR 4.51, 95% CI 1.65–12.34; *p* = 0.003), and early transient temperature rise also showed an elevated estimate (adjusted OR 3.18, 95% CI 1.08–9.31; *p* = 0.035). The delayed-fever estimate did not reach statistical significance (adjusted OR 2.93, 95% CI 0.92–9.40; *p* = 0.070). The exploratory ordered trend was associated with mortality (adjusted OR 1.55, 95% CI 1.14–2.10; *p* = 0.005).

For post-exposure seizure from 72 h through day 7, the overall trajectory-class test was not statistically significant (global *p* = 0.126). The persistent-hyperthermia versus stable-normothermia contrast was positive but imprecise (adjusted OR 2.52, 95% CI 0.93–6.84; *p* = 0.071). The exploratory ordered-trend estimate was 1.43 per class increase (95% CI 1.03–2.00; *p* = 0.035).

Among the 223 patients in the day-8 risk set, persistent hyperthermia versus non-persistent trajectories yielded an exploratory adjusted OR of 3.16 (95% CI 1.07–9.35; *p* = 0.037) for seizure during days 8–90. The exploratory ordered-trend estimate was 1.81 per class increase (95% CI 1.12–2.93; *p* = 0.016). With only 17 events, non-systematic EEG assessment, and competing mortality during follow-up, these estimates were considered hypothesis-generating rather than definitive (Table 3 and Figure 3).

**Table 3:** Adjusted associations between model-based temperature trajectory classes and clinical outcomes.

Outcome	Exposure contrast	Adjusted OR (95% CI)	*p-*value
90-day unfavorable outcome (mRS 3–6)	Global trajectory-class test	—	0.088
90-day unfavorable outcome (mRS 3–6)	Early transient temperature rise vs. stable normothermia	2.00 (0.84–4.76)	0.119
90-day unfavorable outcome (mRS 3–6)	Delayed fever vs. stable normothermia	2.60 (0.90–7.54)	0.079
90-day unfavorable outcome (mRS 3–6)	Persistent hyperthermia vs. stable normothermia	2.36 (1.07–5.20)	0.032
90-day unfavorable outcome (mRS 3–6)	Exploratory ordered trend	1.35 (1.04–1.75)	0.023
90-day mortality	Global trajectory-class test	—	0.019
90-day mortality	Early transient temperature rise vs. stable normothermia	3.18 (1.08–9.31)	0.035
90-day mortality	Delayed fever vs. stable normothermia	2.93 (0.92–9.40)	0.070
90-day mortality	Persistent hyperthermia vs. stable normothermia	4.51 (1.65–12.34)	0.003
90-day mortality	Exploratory ordered trend	1.55 (1.14–2.10)	0.005
Post-exposure seizure (72 h to day 7)	Global trajectory-class test	—	0.126
Post-exposure seizure (72 h to day 7)	Early transient temperature rise vs. stable normothermia	0.73 (0.20–2.67)	0.640
Post-exposure seizure (72 h to day 7)	Delayed fever vs. stable normothermia	1.55 (0.44–5.43)	0.497
Post-exposure seizure (72 h to day 7)	Persistent hyperthermia vs. stable normothermia	2.52 (0.93–6.84)	0.071
Post-exposure seizure (72 h to day 7)	Exploratory ordered trend	1.43 (1.03–2.00)	0.035
Exploratory seizure during days 8–90 after stroke	Persistent hyperthermia vs. non-persistent trajectories (exploratory)	3.16 (1.07–9.35)	0.037
Exploratory seizure during days 8–90 after stroke	Exploratory ordered trend	1.81 (1.12–2.93)	0.016

Global *p*-values are likelihood-ratio tests for the categorical trajectory variable. Stable normothermia was the reference group, except in the exploratory seizure analysis during days 8–90, in which persistent hyperthermia was compared with non-persistent trajectories. Because the global trajectory-class tests for unfavorable functional outcome and post-exposure seizure were not statistically significant, individual class-specific contrasts for these outcomes should be interpreted as exploratory. Ordered-trend and seizure analyses were exploratory. Primary models used baseline and procedural covariates and included onset-to-recanalization or procedure-end time. CI, confidence interval; OR, odds ratio.

**Figure 3:**
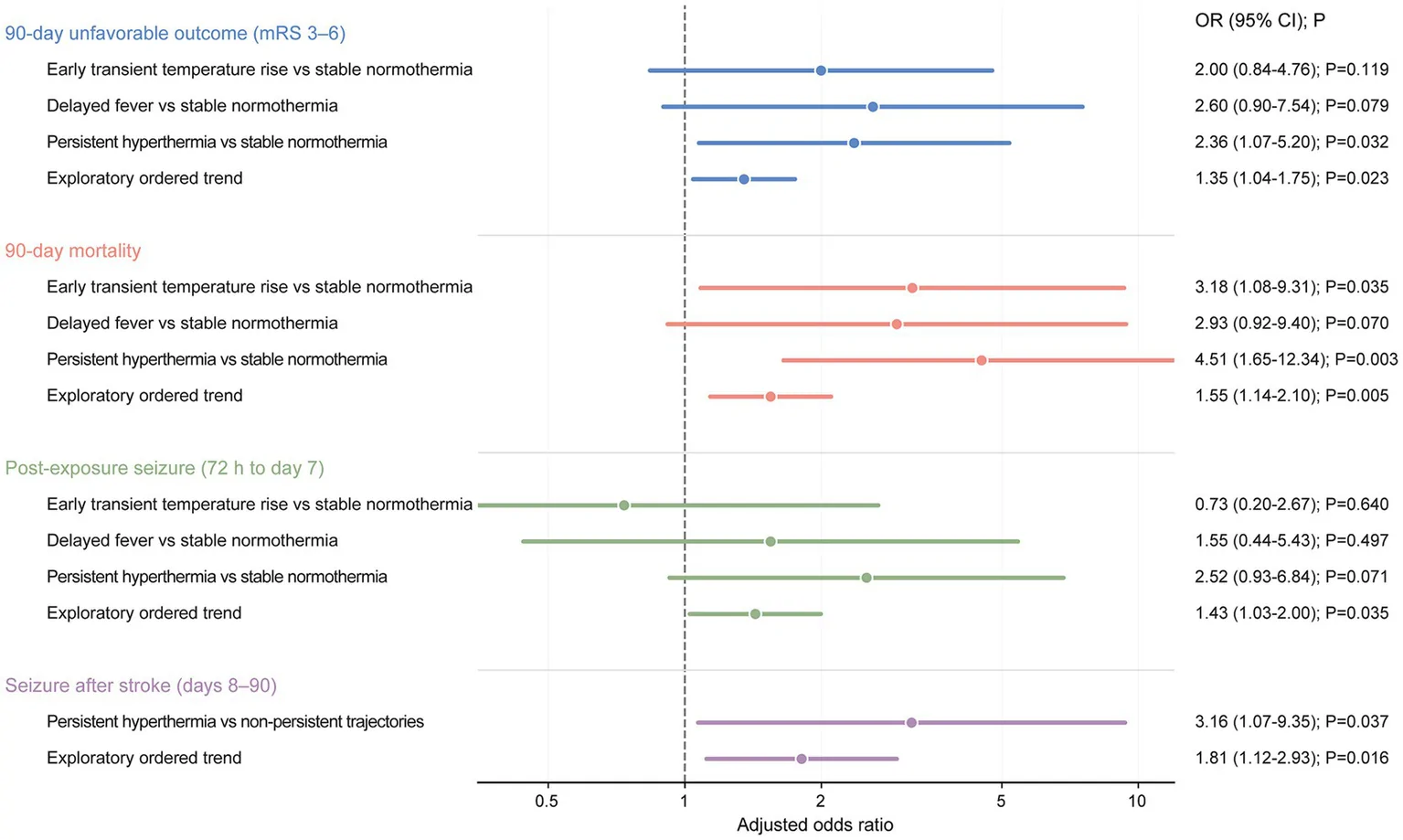
Adjusted associations between temperature trajectories and clinical outcomes. Points and horizontal lines show adjusted ORs and 95% CIs. Stable normothermia was the reference class for categorical analyses, except for seizure after stroke (days 8–90), for which persistent hyperthermia was compared with non-persistent trajectories in the day-8 risk set (*n* = 223). Global trajectory-class *p-*values are reported in Table 3 and the Results. Class-specific contrasts for unfavorable functional outcome and post-exposure seizure are exploratory because their global tests were not significant. Ordered-trend and seizure analyses were exploratory. CI, confidence interval; OR, odds ratio.

### 3.5 Continuous temperature-burden metrics

Continuous temperature-burden metrics were generally consistent in direction with the trajectory analyses (Supplementary Table S3 and Figure 4). In exploratory analyses, peak temperature was associated with unfavorable functional outcome, 90-day mortality, and seizure during days 8–90.

**Figure 4:**
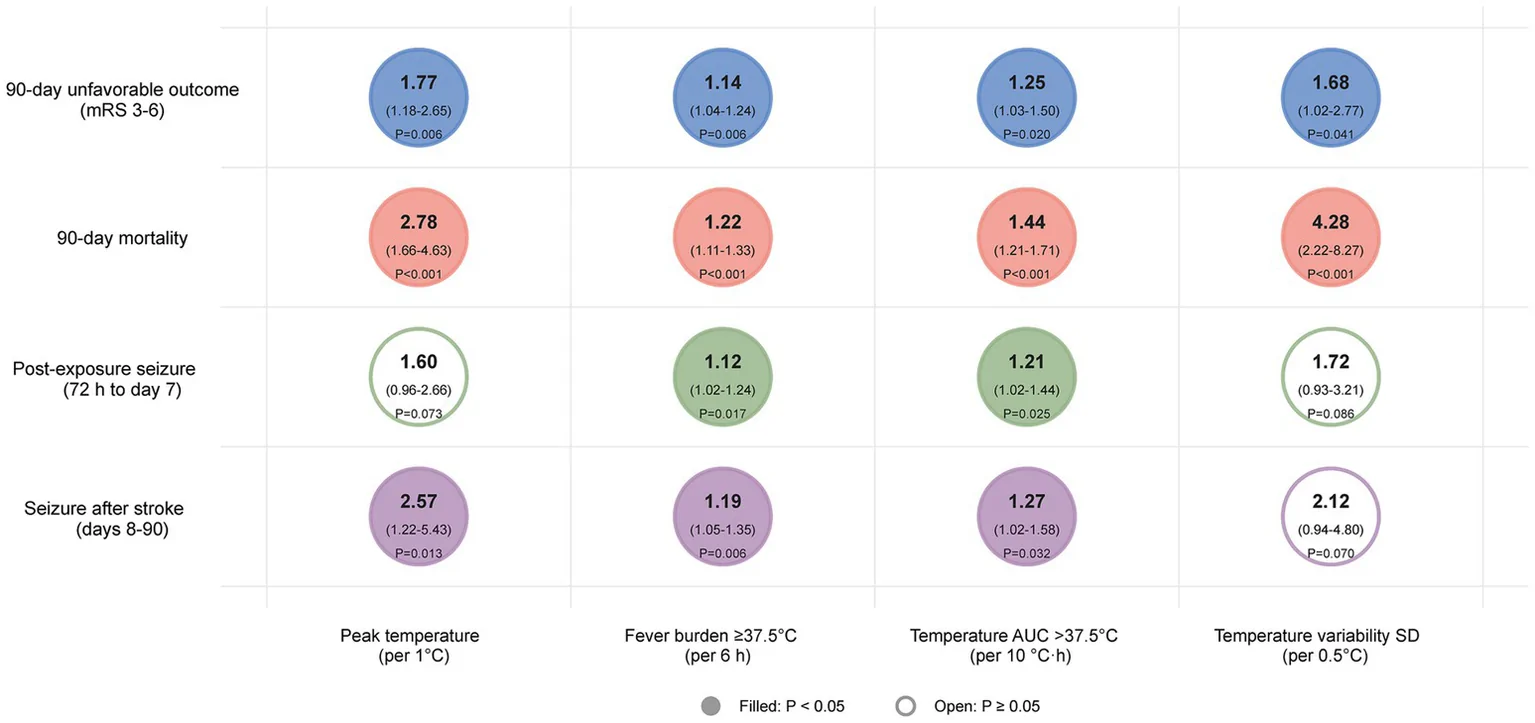
Continuous temperature-burden metrics and clinical outcomes. Circles show adjusted ORs with 95% CIs. Filled circles denote *p* < 0.05 and open circles denote *p* ≥ 0.05. Exposure scales are specified below each metric; OR magnitudes should not be compared directly across metrics. All seizure-related analyses were exploratory; day 8–90 estimates used the day-8 risk set (*n* = 223). *p-*values were unadjusted for multiplicity. CI, confidence interval; OR, odds ratio.

Fever burden and temperature AUC above 37.5 °C were associated with all four outcomes. In the day-8 risk set, temperature AUC showed an exploratory association with seizure during days 8–90 (adjusted OR 1.27 per 10 °C·h, 95% CI 1.02–1.58; *p* = 0.032). Temperature variability was associated with unfavorable outcome and mortality but showed no clear association with seizure outcomes. Because the metrics were scaled differently, their ORs should not be compared directly; all seizure-related continuous analyses remain exploratory.

### 3.6 Sensitivity and subgroup analyses

Sensitivity analyses are summarized in Table 4. For 90-day mortality, adjustment for early antipyretic use attenuated the persistent-hyperthermia estimate from 4.51 to 3.02 (95% CI 0.98–9.32; *p* = 0.055), and the global trajectory-class test was no longer significant (*p* = 0.241). Excluding patients with infection within 72 h yielded an adjusted OR of 3.92 (95% CI 1.18–12.97; *p* = 0.025), with a global *p* = 0.066. Other sensitivity estimates remained in the same direction. The mortality estimate was 4.63 (95% CI 1.69–12.70; *p* = 0.003; global *p* = 0.017) when ischemic core was entered categorically and 3.84 (95% CI 1.50–9.85; *p* = 0.005; global *p* = 0.009) when core was omitted. Alternative handling of core also yielded similar class-specific estimates for unfavorable functional outcome, although the global *p-*values varied (Table 4). For that outcome, the primary global test was not statistically significant, and class-specific estimates were less stable across sensitivity analyses. Antipyretic adjustment attenuated the persistent-hyperthermia estimate from 2.36 to 1.93 (95% CI 0.76–4.91; *p* = 0.168), and exclusion of patients with early infection also attenuated the functional-outcome estimate. Exploratory ordered-trend sensitivity analyses are provided in Supplementary Table S8. Subgroup analyses by age, successful recanalization, and circulation territory showed no evidence of interaction (Supplementary Table S4).

**Table 4:** Sensitivity analyses of the categorical temperature-trajectory associations with 90-day outcomes.

Outcome	Sensitivity analysis	Global trajectory-class *p*	Persistent hyperthermia vs stable normothermia, adjusted OR (95% CI)	*p-*value
90-day unfavorable outcome (mRS 3–6)	Primary categorical analysis (reference)	0.088	2.36 (1.07–5.20)	0.032
90-day unfavorable outcome (mRS 3–6)	Additional adjustment for mechanical ventilation and infection within 72 h	0.154	2.24 (1.01–4.98)	0.047
90-day unfavorable outcome (mRS 3–6)	Additional adjustment for active temperature management	0.129	2.31 (1.04–5.12)	0.039
90-day unfavorable outcome (mRS 3–6)	Additional adjustment for early antipyretic use	0.367	1.93 (0.76–4.91)	0.168
90-day unfavorable outcome (mRS 3–6)	Additional adjustment for physical cooling	0.183	2.26 (0.92–5.59)	0.076
90-day unfavorable outcome (mRS 3–6)	Additional adjustment for early neurological deterioration	0.116	2.26 (1.02–5.02)	0.045
90-day unfavorable outcome (mRS 3–6)	Additional adjustment for pre-stroke mRS	0.087	2.36 (1.07–5.18)	0.033
90-day unfavorable outcome (mRS 3–6)	Exclude patients with infection within 72 h	0.668	1.61 (0.67–3.87)	0.287
90-day unfavorable outcome (mRS 3–6)	Anterior-circulation cohort with ASPECTS adjustment	0.237	2.40 (0.99–5.84)	0.054
90-day unfavorable outcome (mRS 3–6)	Exclude class assignments with posterior probability <0.80	0.095	2.37 (1.02–5.51)	0.045
90-day unfavorable outcome (mRS 3–6)	Ischemic core categorized as 0, >0–30, and >30 mL	0.079	2.45 (1.11–5.41)	0.027
90-day unfavorable outcome (mRS 3–6)	Ischemic core omitted from adjustment set	0.010	2.09 (1.00–4.36)	0.051
90-day mortality	Primary categorical analysis (reference)	0.019	4.51 (1.65–12.34)	0.003
90-day mortality	Additional adjustment for mechanical ventilation and infection within 72 h	0.036	4.28 (1.54–11.85)	0.005
90-day mortality	Additional adjustment for active temperature management	0.021	4.60 (1.66–12.72)	0.003
90-day mortality	Additional adjustment for early antipyretic use	0.241	3.02 (0.98–9.32)	0.055
90-day mortality	Additional adjustment for physical cooling	0.037	4.75 (1.55–14.56)	0.006
90-day mortality	Additional adjustment for early neurological deterioration	0.018	4.55 (1.65–12.55)	0.003
90-day mortality	Additional adjustment for pre-stroke mRS	0.017	4.59 (1.67–12.62)	0.003
90-day mortality	Exclude patients with infection within 72 h	0.066	3.92 (1.18–12.97)	0.025
90-day mortality	Anterior-circulation cohort with ASPECTS adjustment	0.027	4.79 (1.49–15.43)	0.009
90-day mortality	Exclude class assignments with posterior probability <0.80	0.013	4.95 (1.78–13.76)	0.002
90-day mortality	Ischemic core categorized as 0, >0–30, and >30 mL	0.017	4.63 (1.69–12.70)	0.003
90-day mortality	Ischemic core omitted from adjustment set	0.009	3.84 (1.50–9.85)	0.005

The primary categorical model included all four trajectory classes, with stable normothermia as the reference class. The table reports the persistent-hyperthermia versus stable-normothermia contrast together with the likelihood-ratio *p*-value for the overall categorical trajectory term. Each sensitivity model used the outcome-specific primary covariate set and added the indicated variable(s), except that the anterior-circulation analysis omitted the circulation indicator and additionally adjusted for ASPECTS. Early clinical and treatment-response variables were examined only in sensitivity analyses because they occurred during or after formation of the 0–72-h trajectory. Because the primary global trajectory-class test for unfavorable functional outcome was not statistically significant, its class-specific sensitivity estimates should be interpreted as exploratory. CI, confidence interval; END, early neurological deterioration; OR, odds ratio. In additional core sensitivity analyses, ischemic core was entered as 0, >0–30, and >30 mL or omitted from the adjustment set.

## 4 Discussion

In this single-center ICU cohort, body temperature from EVT initiation through 72 h followed four distinct patterns. Evidence for an overall trajectory-class association was clearest for 90-day mortality, whereas the global tests for unfavorable functional outcome and post-exposure seizure were not statistically significant. Persistent hyperthermia had the largest class-specific estimates for both mortality and unfavorable functional outcome, but only the mortality result was supported by a statistically significant global trajectory-class test. The functional-outcome contrast and all seizure-related findings should therefore be viewed as hypothesis-generating, given the non-significant global tests, multiple comparisons, imprecision, and small event counts. The mortality finding is consistent with previous evidence linking even mild temperature elevation after endovascular reperfusion and post-stroke pyrexia with greater ischemic injury and worse outcomes (16, 17). However, the mortality association was attenuated after adjustment for early antipyretic use and after exclusion of patients with early infection.

By preserving information on when temperature increased and whether it subsequently resolved or persisted, trajectory analysis can describe temporal patterns that are not represented by single measurements. Prior stroke studies have shown that acute body temperature and in-hospital physiological dynamics are associated with clinical outcomes, and trajectory approaches have been applied to repeated ICU measurements in stroke populations (18–20). The present analyses did not establish added prognostic value of trajectory classification over simpler temperature metrics such as fever burden or temperature AUC; the class-specific median fever burdens (0, 12.0, 24.6, and 34.0 h) indicate substantial overlap between trajectory class and cumulative fever exposure. Methodological guidance supports latent trajectory approaches when longitudinal measurements may contain heterogeneous temporal patterns that are not adequately summarized by a single average trajectory (21). In this cohort, the trajectory analysis should therefore be viewed as descriptive and exploratory rather than as a new prognostic tool.

In the primary mortality model, persistent hyperthermia had the largest class-specific estimate. The estimate remained elevated when ischemic core was entered categorically or omitted from the model, but it was less robust after adjustment for early antipyretic use and exclusion of early infection. Poor outcome despite angiographic reperfusion is multifactorial, with age, stroke severity, treatment delay, imaging burden, and other clinical factors contributing to futile recanalization (22). Fever has been associated with worse outcomes in patients with non-anoxic acute brain injury (23), and elevated temperature may exacerbate neuroinflammation and blood–brain barrier disruption (24, 25). Persistent hyperthermia may therefore mark ongoing secondary brain injury, systemic inflammation, infection, impaired thermoregulation, or other critical illness rather than acting as an isolated causal exposure.

The imaging profile of the persistent-hyperthermia class did not parallel its clinical severity: this group had a median ischemic core volume of 0 mL, a high median ASPECTS among patients with anterior-circulation stroke, and the lowest rate of symptomatic intracranial hemorrhage despite higher NIHSS and mortality. The same core-volume pattern remained when the analysis was restricted to anterior-circulation stroke. Given the high proportion of software-reported 0-mL estimates and their discordance with clinical severity, measurement error or underestimation by automated CTP processing cannot be excluded; a reported 0 mL should therefore not be interpreted as proof that no ischemic core was present. We accordingly avoided attributing the persistent-hyperthermia phenotype to baseline core volume, and the mortality association remained similar when core was omitted or handled differently in sensitivity analyses.

The seizure analyses were exploratory by design. The first outcome was restricted to events from 72 h through day 7 to avoid overlap with the 0–72-h temperature exposure and should be interpreted as a post-exposure seizure outcome rather than the full conventional acute symptomatic seizure period. The second outcome covered only days 8–90 and should not be equated with long-term post-stroke epilepsy or the broader concept of late seizure. Its risk set comprised 223 patients with documented survival and evaluable follow-up at day 8; patients who died in hospital or were discharged in a moribund condition before day 8 were excluded. Several factors that may coexist with persistent hyperthermia—including greater stroke severity, cortical involvement, hemorrhagic transformation, inflammation, and early seizures—are established or plausible contributors to post-stroke epileptogenesis (26–28). Continuous electroencephalography is recommended in selected critically ill patients when nonconvulsive seizures are suspected, but it was not systematically used in this cohort (29). Only 17 events occurred during days 8–90, and death during this interval was a competing event. These findings are therefore best regarded as hypothesis-generating rather than as support for a temperature-based seizure-prediction rule.

The continuous analyses were broadly consistent with the trajectory findings and suggest that both temperature magnitude and duration may carry prognostic information. Peak temperature and fever burden were associated with several outcomes, and temperature AUC above 37.5 °C was associated with all four outcomes, including an exploratory association with seizure during days 8–90 in the day-8 risk set. Previous studies have linked an early rise in body temperature after stroke and elevated temperature in neurocritical care with unfavorable outcomes, mortality, and longer length of stay (30, 31). Conceptually, trajectory classes emphasize timing and persistence, whereas continuous metrics quantify cumulative temperature elevation.

Early infection, mechanical ventilation, END, and temperature-management measures were not included in the primary models because they could occur during or after trajectory formation. They were instead examined in sensitivity models to assess robustness. The observed trajectories likely reflect both underlying physiological processes and treatment responses. Antipyretic use and physical cooling differed markedly across trajectory classes, and adjustment for early antipyretic use materially attenuated both the mortality and functional-outcome estimates. Because antipyretic treatment was initiated in response to evolving temperature during the same exposure window, conventional adjustment cannot cleanly separate the underlying temperature course from treatment effects and may be affected by confounding by indication and mediation. Accordingly, these classes should be viewed as temperature patterns observed under routine care, not as untreated biological trajectories. Guidelines and interventional studies indicate that temperature management is a multicomponent strategy and that antipyretic or device-based fever-control approaches can reduce temperature or fever burden without consistently improving functional outcomes (32–35). A systematic review of acute brain injury similarly found that evidence for clinical outcome benefit remains limited and context-dependent (36). These considerations argue against a causal interpretation of our observational associations.

These findings do not imply that all patients with peri-procedural or early post-EVT fever should receive aggressive cooling, and they do not demonstrate that lowering temperature improves outcome. General neurocritical care guidance emphasizes prevention of secondary brain injury and early recognition of complications after ischemic stroke (37, 38). Fever should prompt evaluation for infectious and noninfectious causes because antipyretic therapy alone may be insufficient and fever may serve as a marker of underlying complications (39). Persistent hyperthermia may therefore identify patients who warrant closer neurological assessment, evaluation for infection or hemorrhagic complications, and consideration of seizure monitoring.

Several limitations should be considered. First, this retrospective single-center cohort was restricted to patients selected for ICU care after EVT, and ICU disposition was partly influenced by bed availability, limiting generalizability and leaving residual confounding possible. Second, the exposure window began at arterial puncture and included both intra-procedural and early post-procedural influences. Requiring eligible temperature measurements at all nine prespecified points through 72 h conditioned inclusion on survival and continued observation; some patients were excluded because of early deterioration or death, early ICU transfer, self-discharge, or absence of a measurement within a prespecified ±2-h window. The direction of this selection bias, particularly for mortality, is uncertain. Third, ischemic core was derived with Dr. Wise-CTP using an rCBF <30% threshold. Nearly half of the cohort had software-reported 0-mL estimates, and measurement error or underestimation cannot be excluded. The available technical records did not specify a minimum lesion-size or cluster-size threshold, and the rCBF <30% threshold is less well established in posterior-circulation stroke. However, anterior-circulation analyses with ASPECTS adjustment and alternative handling or omission of core yielded similar mortality estimates. Fourth, the four-class structure was cohort-derived and requires external validation. Fifth, the global trajectory-class test for the primary functional outcome was not significant, and the mortality association was attenuated in key sensitivity analyses; secondary and exploratory analyses were not adjusted for multiplicity. Finally, seizure ascertainment was limited by non-systematic EEG use, small event counts, selection into the day-8 risk set, and competing mortality (40).

## 5 Conclusion

Peri-procedural and early post-EVT temperature trajectories were heterogeneous in ICU patients with acute ischemic stroke. Greater temperature exposure was associated with poorer 90-day outcomes, consistent with previous work on post-stroke fever. Persistent hyperthermia was associated with 90-day mortality in the primary analysis, but this association was attenuated in key sensitivity analyses. In this cohort, we did not establish added prognostic value of trajectory classification over simpler temperature metrics such as fever burden and temperature AUC. We therefore view the trajectory analysis as confirmatory of the broader fever–outcome association and methodologically exploratory, rather than as a new prognostic tool.

## Data Availability

The datasets presented in this article are not readily available. De-identified data and the analytic code used in this study are available from the corresponding author upon reasonable request, subject to institutional and ethical restrictions. Requests to access the datasets should be directed to XW, wxb49752403@163.com.
