# Supplementary material for: Peri-procedural and early body temperature trajectories after endovascular thrombectomy and clinical outcomes in intensive care unit patients with acute ischemic stroke: a retrospective cohort study
Source: Front Neurol. 2026 Sep 25;17:1941980. doi: 10.3389/fneur.2026.1941980 (PMC13649389; doi:10.3389/fneur.2026.1941980)
Supplement: Supplementary file 1 [file Supplementary_file_1.DOCX]

**Supplementary Material**

**Supplementary Methods: Temperature trajectory identification**

Temperature trajectories from EVT initiation through 72 hours were identified from axillary temperature measurements at 0, 2, 6, 12, 24, 36, 48, 60, and 72 hours. Routine clinical temperature measurements were more frequent than the nine values selected for analysis. The grid was prespecified to capture rapid peri-procedural changes more densely during the first 12–24 hours, followed by 12-hour sampling through 72 hours. A measurement obtained within 2 hours before or after each prespecified time point was eligible, and the measurement closest to the target time was selected when more than one was available. Patients were required to have eligible measurements at all nine time points. Because the early portion of the grid was sampled more densely, the fitted trajectories may be more sensitive to transient peri-procedural changes than to changes occurring later in the exposure window. Because inclusion required one eligible measurement at every prespecified point, frequent routine monitoring did not necessarily produce a complete nine-point series.

For each patient, a cubic polynomial function of time summarized the individual 0- to 72-hour curve. Measurement time was divided by 72 hours to scale it to the 0–1 interval. The patient-specific intercept, linear, quadratic, and cubic coefficients were standardized across patients using StandardScaler and entered into Gaussian mixture models with one to five candidate classes. Models were fitted with the GaussianMixture implementation in scikit-learn using unrestricted full covariance matrices, 10 initializations, covariance regularization of 1×10⁻⁵, a maximum of 500 expectation–maximization iterations, and a fixed random seed. Candidate solutions were evaluated using the Akaike and Bayesian information criteria, entropy, minimum class proportion, average posterior probability, convergence, and clinical interpretability.

The four-class solution was retained because it yielded adequately sized, clinically interpretable classes with high posterior assignment probabilities. The five-class solution had a lower AIC and a marginally lower BIC than the four-class solution (BIC 147.7 vs 149.1), but its smallest class contained only one patient (0.4% of the cohort). Classification uncertainty was summarized using average and median posterior probabilities and the number of patients with maximum posterior probability <0.80. In a post hoc initialization-sensitivity analysis, the four- and five-class models were refitted across 30 random-state values using the same model specification and 10 initializations per fit. The four-class partition used in the primary analysis was reproduced exactly in 28/30 fits; five-class solutions were more sensitive to initialization and produced a class of five or fewer patients in 11/30 fits (Supplementary Table S7).

**Supplementary Methods: Selection-bias analyses**

To assess potential selection into ICU care and the final analytic cohort, screened EVT patients were compared according to post-EVT ICU admission and analytic inclusion status. Continuous and ordinal variables were compared using Mann–Whitney U tests, and categorical variables using Pearson chi-square or Fisher exact tests, as appropriate. Signed standardized mean differences were calculated as the first group minus the second group. Reasons for incomplete 72-hour temperature observation were reviewed from the source records and summarized separately.

**Supplementary Table**

**Supplementary Table S1. Model selection for temperature trajectories**

| **Number of classes** | **AIC** | **BIC** | **Entropy** | **Minimum class proportion** | **Minimum average posterior probability** | **Converged** | **Iterations** |
| --- | --- | --- | --- | --- | --- | --- | --- |
| 1 | 560.3 | 608.8 | NA | 1.000 | 1.000 | Yes | 2 |
| 2 | 249.0 | 349.6 | 0.889 | 0.489 | 0.955 | Yes | 13 |
| 3 | 132.5 | 285.1 | 0.867 | 0.228 | 0.910 | Yes | 18 |
| 4 | −55.5 | 149.1 | 0.917 | 0.135 | 0.909 | Yes | 18 |
| 5 | −109.0 | 147.7 | 0.930 | 0.004 | 0.912 | Yes | 26 |

AIC, Akaike information criterion; BIC, Bayesian information criterion; NA, not applicable.

**Supplementary Table S2. Diagnostics for the selected four-class model**

| **Trajectory class** | **n** | **Proportion (%)** | **Average posterior probability** | **Median posterior probability** | **n with posterior probability <0.80** |
| --- | --- | --- | --- | --- | --- |
| Stable normothermia | 89 | 37.6 | 0.974 | 0.998 | 5 |
| Early transient temperature rise | 49 | 20.7 | 0.973 | 1.000 | 2 |
| Delayed fever | 32 | 13.5 | 0.988 | 0.995 | 0 |
| Persistent hyperthermia | 67 | 28.3 | 0.909 | 0.943 | 10 |

Posterior probabilities refer to each patient’s maximum probability of assignment to the selected class.

**Supplementary Table S3. Continuous temperature-burden metrics and clinical outcomes**

| **Outcome** | **Exposure** | **Adjusted OR (95% CI)** | **P value** |
| --- | --- | --- | --- |
| 90-day unfavorable outcome (mRS 3–6) | Peak temperature, per 1°C | 1.77 (1.18–2.65) | 0.006 |
| 90-day unfavorable outcome (mRS 3–6) | Fever burden ≥37.5°C, per 6 h | 1.14 (1.04–1.24) | 0.006 |
| 90-day unfavorable outcome (mRS 3–6) | Temperature AUC above 37.5°C, per 10 °C·h | 1.25 (1.03–1.50) | 0.020 |
| 90-day unfavorable outcome (mRS 3–6) | Temperature variability SD, per 0.5°C | 1.68 (1.02–2.77) | 0.041 |
| 90-day mortality | Peak temperature, per 1°C | 2.78 (1.66–4.63) | <0.001 |
| 90-day mortality | Fever burden ≥37.5°C, per 6 h | 1.22 (1.11–1.33) | <0.001 |
| 90-day mortality | Temperature AUC above 37.5°C, per 10 °C·h | 1.44 (1.21–1.71) | <0.001 |
| 90-day mortality | Temperature variability SD, per 0.5°C | 4.28 (2.22–8.27) | <0.001 |
| Post-exposure seizure (72 h to day 7) | Peak temperature, per 1°C | 1.60 (0.96–2.66) | 0.073 |
| Post-exposure seizure (72 h to day 7) | Fever burden ≥37.5°C, per 6 h | 1.12 (1.02–1.24) | 0.017 |
| Post-exposure seizure (72 h to day 7) | Temperature AUC above 37.5°C, per 10 °C·h | 1.21 (1.02–1.44) | 0.025 |
| Post-exposure seizure (72 h to day 7) | Temperature variability SD, per 0.5°C | 1.72 (0.93–3.21) | 0.086 |
| Seizure during days 8–90 after stroke | Peak temperature, per 1°C | 2.57 (1.22–5.43) | 0.013 |
| Seizure during days 8–90 after stroke | Fever burden ≥37.5°C, per 6 h | 1.19 (1.05–1.35) | 0.006 |
| Seizure during days 8–90 after stroke | Temperature AUC above 37.5°C, per 10 °C·h | 1.27 (1.02–1.58) | 0.032 |
| Seizure during days 8–90 after stroke | Temperature variability SD, per 0.5°C | 2.12 (0.94–4.80) | 0.070 |

Models used the same outcome-specific baseline and procedural covariates as the trajectory analyses. The post-exposure seizure outcome begins at 72 hours to avoid overlap with the temperature exposure window and does not cover the full conventional acute symptomatic seizure period. Models for seizure during days 8–90 included 223 patients with documented survival and evaluable follow-up at the beginning of day 8; patients who died in hospital or were discharged in a moribund condition before day 8 were excluded. All seizure-related continuous analyses were exploratory. CI, confidence interval; OR, odds ratio.

**Supplementary Table S4. Exploratory subgroup analyses for 90-day unfavorable functional outcome**

| **Subgroup** | **Level** | **N** | **Events** | **Ordinal trajectory OR (95% CI)** | **P for interaction** |
| --- | --- | --- | --- | --- | --- |
| Successful recanalization | Yes | 187 | 109 | 1.34 (1.01–1.78) | 0.971 |
| Successful recanalization | No | 50 | 34 | 1.31 (0.61–2.80) | 0.971 |
| Age ≥75 years | Yes | 81 | 58 | 1.28 (0.77–2.12) | 0.656 |
| Age ≥75 years | No | 156 | 85 | 1.38 (1.02–1.87) | 0.656 |
| Anterior circulation | Yes | 186 | 111 | 1.32 (0.99–1.76) | 0.712 |
| Anterior circulation | No | 51 | 32 | 1.66 (0.85–3.25) | 0.712 |

Subgroup analyses used the exploratory ordered trajectory variable and were limited to baseline or procedural characteristics. The subgroup-defining variable was excluded from the corresponding adjustment set. CI, confidence interval; OR, odds ratio.

**Supplementary Table S5. Characteristics of screened EVT patients according to ICU admission and analytic inclusion**

| **Variable** | **ICU-admitted (n=336)** | **Non-ICU (n=132)** | **Signed SMD** | **P value** |
| --- | --- | --- | --- | --- |
| Panel A: ICU-admitted versus non-ICU EVT patients |  |  |  |  |
| Age, years | 70 (61–79) | 66 (57.8–75) | 0.252 | 0.012 |
| Male sex | 231/336 (68.8%) | 98/132 (74.2%) | −0.122 | 0.242 |
| Baseline NIHSS | 18 (13–23) | 12 (8–15) | 1.002 | <0.001 |
| Pre-stroke mRS | 0 (0–1) | 0 (0–1) | −0.008 | 0.510 |
| Anterior circulation | 257/336 (76.5%) | 113/132 (85.6%) | −0.234 | 0.029 |
| General anesthesia | 317/336 (94.3%) | 93/132 (70.5%) | 0.661 | <0.001 |
| Mechanical ventilation | 204/336 (60.7%) | 23/132 (17.4%) | 0.990 | <0.001 |
| Successful recanalization | 253/336 (75.3%) | 105/132 (79.5%) | −0.102 | 0.329 |
| sICH | 31/336 (9.2%) | 5/132 (3.8%) | 0.222 | 0.047 |
| In-hospital mortality | 26/336 (7.7%) | 5/132 (3.8%) | 0.170 | 0.122 |
| 90-day mortality | 83/313 (26.5%) | 12/132 (9.1%) | 0.468 | <0.001 |
| Panel B: Included versus excluded ICU patients |  |  |  |  |
| Age, years | 70 (60–78) | 72 (61.5–79) | −0.124 | 0.416 |
| Male sex | 163/237 (68.8%) | 68/99 (68.7%) | 0.002 | 0.987 |
| Baseline NIHSS | 18 (12–22) | 19 (15–24) | −0.316 | 0.010 |
| Pre-stroke mRS | 0 (0–1) | 0 (0–1) | −0.276 | 0.036 |
| Anterior circulation | 186/237 (78.5%) | 71/99 (71.7%) | 0.157 | 0.183 |
| General anesthesia | 228/237 (96.2%) | 89/99 (89.9%) | 0.250 | 0.023 |
| Mechanical ventilation | 138/237 (58.2%) | 66/99 (66.7%) | −0.175 | 0.149 |
| Successful recanalization | 187/237 (78.9%) | 66/99 (66.7%) | 0.278 | 0.018 |
| sICH | 22/237 (9.3%) | 9/99 (9.1%) | 0.007 | 0.956 |
| In-hospital mortality | 9/237 (3.8%) | 17/99 (17.2%) | −0.447 | <0.001 |
| 90-day mortality | 60/237 (25.3%) | 23/76 (30.3%) | −0.111 | 0.395 |
| Panel C: Included versus patients excluded for incomplete temperature observations |  |  |  |  |
| Age, years | 70 (60–78) | 70 (61–79) | −0.064 | 0.728 |
| Male sex | 163/237 (68.8%) | 39/60 (65.0%) | 0.080 | 0.575 |
| Baseline NIHSS | 18 (12–22) | 20.5 (16–27) | −0.526 | <0.001 |
| Pre-stroke mRS | 0 (0–1) | 0 (0–1) | −0.329 | 0.069 |
| Anterior circulation | 186/237 (78.5%) | 43/60 (71.7%) | 0.158 | 0.262 |
| General anesthesia | 228/237 (96.2%) | 54/60 (90.0%) | 0.247 | 0.050 |
| Mechanical ventilation | 138/237 (58.2%) | 44/60 (73.3%) | −0.322 | 0.032 |
| Successful recanalization | 187/237 (78.9%) | 43/60 (71.7%) | 0.168 | 0.231 |
| sICH | 22/237 (9.3%) | 6/60 (10.0%) | −0.024 | 0.865 |
| Death within 72 h | 0/237 (0.0%) | 11/60 (18.3%) | −0.670 | <0.001 |
| In-hospital mortality | 9/237 (3.8%) | 12/60 (20.0%) | −0.517 | <0.001 |
| 90-day mortality | 60/237 (25.3%) | 17/60 (28.3%) | −0.068 | 0.634 |

Data are presented as median (IQR) or n/N (%). Signed standardized mean differences (SMDs) were calculated as the first group minus the second group. P values were obtained using Mann–Whitney U tests for continuous or ordinal variables and Pearson chi-square tests for categorical variables. Available-case denominators were used. In the ICU-admitted group, baseline NIHSS and pre-stroke mRS were available for 333/336 patients and 90-day vital status for 313/336. Among the 99 excluded ICU patients, baseline NIHSS and pre-stroke mRS were available for 96/99 and 90-day vital status for 76/99. Mechanical ventilation was defined as invasive ventilatory support continuing after EVT; intraprocedural intubation for general anesthesia was not counted if extubation occurred at the end of the procedure. When ICU beds were unavailable, selected patients requiring continued ventilatory support could be managed in an enhanced-monitoring area of the neurosurgical ward. EVT, endovascular thrombectomy; ICU, intensive care unit; NIHSS, National Institutes of Health Stroke Scale; mRS, modified Rankin Scale; sICH, symptomatic intracranial hemorrhage.

**Supplementary Table S6. Reasons for incomplete 72-hour temperature observation and corresponding clinical characteristics**

| **Reason for incomplete observation** | **n (%)** | **Baseline NIHSS, median (IQR)** | **Mechanical ventilation** | **In-hospital mortality** | **90-day mortality** | **90-day unfavorable outcome** |
| --- | --- | --- | --- | --- | --- | --- |
| Death before completion of 72 h | 11/60 (18.3%) | 31 (28–36.5) | 10/11 (90.9%) | 11/11 (100.0%) | 11/11 (100.0%) | 11/11 (100.0%) |
| Transfer out of ICU before 72 h | 15/60 (25.0%) | 16 (15–18) | 10/15 (66.7%) | 0/15 (0.0%) | 0/15 (0.0%) | 3/15 (20.0%) |
| Self-discharge before 72 h | 9/60 (15.0%) | 19 (17–24) | 7/9 (77.8%) | 0/9 (0.0%) | 5/9 (55.6%) | 9/9 (100.0%) |
| Routine documentation incomplete while hospitalized | 23/60 (38.3%) | 22 (16–24) | 15/23 (65.2%) | 1/23 (4.3%) | 1/23 (4.3%) | 7/23 (30.4%) |
| Other/timing inconsistency | 2/60 (3.3%) | 21 (20.5–21.5) | 2/2 (100.0%) | 0/2 (0.0%) | 0/2 (0.0%) | 1/2 (50.0%) |
| Total | 60/60 (100.0%) |  |  |  |  |  |

Among the nine patients who self-discharged before 72 hours, five were documented as being in a moribund condition at discharge; all five died by day 90. None of the 15 patients transferred out of the ICU before 72 hours died by day 90, and 3/15 (20.0%) had an unfavorable 90-day functional outcome. Data are presented as n/N (%) unless otherwise indicated. ICU, intensive care unit; NIHSS, National Institutes of Health Stroke Scale.

**Supplementary Table S7. Post hoc initialization-sensitivity analysis of the four- and five-class Gaussian mixture models**

| **Metric** | **4-class solution** | **5-class solution** |
| --- | --- | --- |
| Exact reproduction of submitted 4-class patient partition | 28/30 fits (93.3%) | Not applicable |
| Adjusted Rand index vs submitted 4-class partition | Median 1.000; range 0.813–1.000 | Not applicable |
| BIC across random-state values | Median 149.1; range 149.0–198.9 | Median 199.2; range 136.0–273.8 |
| Fits with a very small class (≤5 patients) | 0/30 | 11/30 (36.7%) |
| Minimum extracted class size across fits | 30–32 | 1–32 |

Models were refitted across 30 random-state values using the same specification and 10 initializations per fit. The adjusted Rand index is label invariant; a value of 1 indicates identical patient partitioning despite arbitrary relabeling of mixture components. This post hoc analysis assessed sensitivity to initialization and was not intended as external validation of the trajectory structure. BIC, Bayesian information criterion.

**Supplementary Table S8. Exploratory ordered-trend sensitivity analyses**

| **Outcome** | **Sensitivity analysis** | **Adjusted OR per one-class increase (95% CI)** | **P value** |
| --- | --- | --- | --- |
| 90-day unfavorable outcome | Primary ordered-trend analysis | 1.35 (1.04–1.75) | 0.023 |
| 90-day unfavorable outcome | + Mechanical ventilation and infection within 72 h | 1.32 (1.02–1.72) | 0.035 |
| 90-day unfavorable outcome | + Active temperature management | 1.34 (1.03–1.73) | 0.029 |
| 90-day unfavorable outcome | + Early antipyretic use | 1.25 (0.92–1.70) | 0.150 |
| 90-day unfavorable outcome | + Physical cooling | 1.33 (0.99–1.78) | 0.058 |
| 90-day unfavorable outcome | + END | 1.33 (1.02–1.72) | 0.034 |
| 90-day unfavorable outcome | + Pre-stroke mRS | 1.35 (1.04–1.75) | 0.023 |
| 90-day unfavorable outcome | Exclude infection within 72 h | 1.17 (0.88–1.56) | 0.270 |
| 90-day unfavorable outcome | Anterior circulation + ASPECTS | 1.34 (1.01–1.79) | 0.046 |
| 90-day unfavorable outcome | Posterior probability ≥0.80 | 1.36 (1.03–1.79) | 0.029 |
| 90-day mortality | Primary ordered-trend analysis | 1.55 (1.14–2.10) | 0.005 |
| 90-day mortality | + Mechanical ventilation and infection within 72 h | 1.55 (1.13–2.13) | 0.007 |
| 90-day mortality | + Active temperature management | 1.54 (1.13–2.10) | 0.006 |
| 90-day mortality | + Early antipyretic use | 1.35 (0.96–1.91) | 0.086 |
| 90-day mortality | + Physical cooling | 1.54 (1.10–2.16) | 0.012 |
| 90-day mortality | + END | 1.54 (1.13–2.10) | 0.006 |
| 90-day mortality | + Pre-stroke mRS | 1.55 (1.14–2.10) | 0.005 |
| 90-day mortality | Exclude infection within 72 h | 1.46 (1.01–2.12) | 0.046 |
| 90-day mortality | Anterior circulation + ASPECTS | 1.55 (1.08–2.22) | 0.018 |
| 90-day mortality | Posterior probability ≥0.80 | 1.59 (1.16–2.18) | 0.004 |

The ordered trajectory variable ranked the four classes by increasing fever burden and was modeled per one-class increase. These analyses are exploratory and complement, rather than replace, the categorical trajectory analysis. Adjustment sets and sensitivity restrictions were otherwise identical to those described for revised Table 4. ASPECTS, Alberta Stroke Program Early CT Score; END, early neurological deterioration; OR, odds ratio.

**Supplementary Table S9. Pretreatment ischemic core distribution in anterior-circulation stroke**

| **Trajectory class** | **n** | **Software-reported 0-mL core, n (%)** | **Ischemic core volume, mL, median (IQR)** |
| --- | --- | --- | --- |
| Overall anterior circulation | 186 | 86 (46.2) | 5.0 (0–24.5) |
| Stable normothermia | 70 | 38 (54.3) | 0 (0–20.0) |
| Early transient temperature rise | 41 | 12 (29.3) | 20.0 (0–35.0) |
| Delayed fever | 23 | 7 (30.4) | 8.0 (0–33.5) |
| Persistent hyperthermia | 52 | 29 (55.8) | 0 (0–15.0) |

Among patients with anterior-circulation stroke, ischemic core volume differed across the four trajectory classes (Kruskal–Wallis P=0.002). Recorded 0-mL values were direct software outputs rather than missing or imputed observations.

**Supplementary figure legends**

**Supplementary Figure S1. Study cohort derivation.**

The flowchart shows the sequential selection of the study cohort. Among 468 patients who underwent endovascular thrombectomy during the study period, 132 were excluded because they were not admitted to the ICU after EVT, 60 because of insufficient temperature data during the first 72 h, 23 because of missing 90-day modified Rankin Scale assessments or follow-up data, and 16 because of incomplete key baseline or procedural variables. The final analytic cohort included 237 patients.

**Supplementary Figure S2. Trajectory model selection.**

AIC and BIC are plotted on the left axis, whereas entropy and minimum class proportion are shown on the right axis after multiplication by 100. The five-class solution had a lower AIC and a marginally lower BIC than the selected four-class solution but produced a singleton class (0.4% of the cohort). The four-class solution was retained on the basis of class size, posterior classification, parsimony, stability, and clinical interpretability.

**Supplementary Figure S3. Fever burden distribution across trajectory classes.**

Boxplots and jittered observations show cumulative time at temperature ≥37.5°C during the first 72 hours after EVT initiation. Diamonds indicate means.
